# Prevalence and Associated Factors of Unplanned Re-Laparatomy after Non-Trauma Emergency Laparatomy in Resource-Limited Settings, 2023: A Retrospective Chart Review

**DOI:** 10.4314/ejhs.v34i3.4

**Published:** 2024-05

**Authors:** Megbar Dessalegn, Tilahun Deresse, George Eskandar, Molla Yigzaw Birhanu

**Affiliations:** 1 Department of Surgery, School of Medicine, Debre Markos University; 2 Department of Surgery, School of Medicine, Debre Birhan University; 3 Department of Surgery, Glan Clwyd Hospital, Rhyl, UK; 4 Department of Public Health, College of Health Sciences, Debre Markos University

**Keywords:** Un-planned surgery, Re-laparatomy, emergency surgery, postoperative complications

## Abstract

**Background:**

Emergency laparatomy may need subsequent re-laparatomy which has high rate of mortality. However, reports on rates and associated factors of un-planned re-laparatomy are few. This study aimed to determine the prevalence and associated factors of re-laparotomy after non-trauma emergency laparatomy at Debre Markos Comprehensive Specialized Hospital, Northwest Ethiopia, 2023.

**Methods:**

This was a retrospective chart review conducted at Debre Markos Comprehensive Specialized Hospital in Ethiopia among patients who had undergone emergency laparatomy between January 1, 2019 and December 31, 2022. A sample of 384 individuals were selected using simple random sampling technique. Data were extracted from March 01, 2023, to May 1, 2023, cleaned, entered into Epi-Data version 3.1, and analyzed with STATA version 14.1. Predictor variables with P value < 0.05 in multivariable logistic regression were reported

**Results:**

From 384 patients who had emergency laparatomy, 33(8.6%) needed re-laparatomy in the early post-operative period. All re-laparotomies were unplanned and done during the primary Hospital admission period Patients who were hypotensive preoperatively[AOR: 3.3 (95% CI: (1.88, 9.40))] and with longer operation time (greater than 1 hour) [AOR: 4.5 (95% CI: (1.88, 10.64)] had increased risk for unplanned re-laparatomy.

**Conclusion:**

The re-laparatomy rate in this study was high with higher risk among patients with preoperative hypotension and longer procedure time. The findings emphasize a need for advocacy on preoperative patient resuscitation and monitoring.

## Introduction

Re-laparotomy is defined as any return to the operating room with surgical re-entry of the abdominal cavity. Some studies use a cutoff point of 30 or 60 days after the first operation to classify interventions performed after the first surgery as reoperations while others relate relaparatomy to operations, in addition to the time when they are performed, to the anatomical site as local or regional (1). Relaparotomy after emergency surgery for non-trauma intra-abdominal catastrophes is associated with increased morbidity and mortality (2). This increased risk is higher despite improvements in antibiotic use, intensive care, and surgical treatment (3).

Re-laparatomies are performed under general anesthesia approximately every 24–48 hours in the operating room or bedside in the intensive care unit, regardless of the patient's clinical situation[4]. However, the criteria for performing a re-laparatomy, the appropriate timing, and indication in patients with secondary peritonitis are neither uniform nor precisely defined (5).

A cohort study by Guevara OA, Rubio-Romero JA, and Ruiz-Parra A showed a 5.9% cumulative incidence of unplanned reoperations. Patients operated on in emergency conditions had a 1.79 crude relative risk (RR, 1.79) of reoperation with a significantly higher risk of death (RR, 8.94) and increased hospital stay (1,6). A study by Negussie et al. at Tikur Anbessa Specialized Hospital in Ethiopia on pediatrics re-laparotomies reported a prevalence of 17.2% (7). A relatively higher magnitude (24%) was reported by Scriba et al and Ugumba et al in other African settings with a higher need for intensive care unit admissions, morbidity, and mortality rates [8, 9]. Turrentine FE, et al., also revealed an increase in unplanned readmission from unplanned abdominal reoperations (10).

Unplanned reoperation is an undesirable outcome with considerable risks and an increasingly assessed quality of care metric (11,12). Such unplanned interventions are associated with complications resulting from the progression of the pathology, patient's general health status, or the surgical technique (6)]. In a retrospective study from Ethiopia, it was reported that the most common surgeries that needed re-laparotomy were related to perforated appendicitis (27.1%), bowel obstructions (21.7%), and trauma (13.4%) complicated by intra-abdominal abscess (44.23%), wound dehiscence (13.2%), and anastomotic leak (11.6%) (13). However, a cross-sectional study on the clinical records of pediatric patients undergoing unplanned re-operations in Chile showed that the cause was attributable to the surgical technique or planning (6).

There were only a few published studies (7,8, 13-15) on re-laparatomy from Ethiopia and Africa. This study aimed to analyze the magnitude of unplanned re-laparatomy after emergency laparatomy so as to improve the patient care. Moreover, it could provide baseline data for further study.

## Materials and Methods

**Study design**: This was a hospital-based cross-sectional study.

**Study area and period**: The study was conducted at Debre Markos Comprehensive Specialized Hospital (DMCSH) in Debre Markos City, Northwest Ethiopia. Debre Markos City is located approximately 295 km northwest of Addis Ababa. Debre Markos Comprehensive Specialized Hospital is a teaching hospital with 300 beds serving over five million people. It has 51 specialist physicians, 63 general practitioners, 386 nurses, and other support staff. The Department of Surgery is staffed with 15 surgeons and 18 general practitioners, and 7 anesthetists to deliver elective and emergency surgical services. The surgical team is better organized after 2019. The emergency surgical service has a quarterly performance of 270(75%) emergency procedures. The study was conducted from March 01, 2023 to May 1, 2023 on patients operated on from January 1, 2019 to December 31, 2022.

**Populations**:The source population was patients who had undergone emergency laparatomy at Debre Markos Comprehensive Specialized Hospital. On the other hand, the study population was made up of selected patients who had undergone emergency laparatomy from January 1, 2019 - December 31, 2022 at Debre Markos Comprehensive Specialized Hospital.

**Inclusion and exclusion criteria**: All patients who were admitted and underwent emergency laparatomy between January 1, 2019 and December 31, 2022, at Debre Markos Comprehensive Specialized Hospital were included. However, all cases with simple appendectomy, cholecystectomy, and obstetric laparatomy were excluded from the study as these patients had limited incisions, initially tried on antibiotics as non-operative management or physiologic changes related to pregnancy. Charts of patients who were transferred from other hospitals after surgical interventions or incomplete patient charts (without at least one progress note and discharge summary) were also excluded.

**Sampling**: The list of study units (identified by medical record numbers) that underwent emergency laparatomy was obtained from the health information and management system (HIMS) logbook of the operation theatre at DMCSH. The sample was selected using simple random sampling method. The total sample size was determined using a single-population proportion formula [(Zα/2)^2^P(1-P/d^2^)] based on the assumptions of 95% CI, 5% margin of error, and 50% proportion (P). With an added 10% estimated non-response rate,this yielded a final sample size of 384.

**Variables**: The dependent variable is prevalence of re-laparatomy. The independent variables are patient socio-demographic factors (age, sex, residence, mode of arrival, mode of admission, and referral status), preoperative factors (bloodpressure, pulse rate, fever, abnormal leukocyte count, indication for surgery, duration of symptoms, presence of sepsis, presence of anemia,presence of comorbidity, use of prophylactic antibiotics, previous surgery, ASA status,vasopressor use, blood transfusion, diffuse abdominal tenderness,and serum hemoglobin), intra-variables (use of WHO checklist, duration of operative anesthesia, duration of surgery,blood transfusion,vasopressor use, bowel ischemia, degree of peritoneal contamination, and source of peritoneal contamination),post-operative variables (presence of postoperative complications, need for re-operation, ICU admission, need for re-laparatomy, and intra-abdominal collection).

**Operational definitions**: Preoperative hypotension is blood pressure of less than 90/60 mmHg. Long procedure time is a surgical procedure time greater than one hour.

**Data collection**: A data extraction checklist was prepared based on the literature reviewed (, 6, 7, 9, 13) and used to extract necessary data from the charts of the study participants taken from the archive of Debre Markos Comprehensive Specialized Hospital. It contained the following four sections: socio-demographic data, preoperative clinical data, intraoperative clinical data, and post-operative follow-up data. Data were collected by four nurses.

**Data quality assurance**: The data extraction checklist was evaluated by subject matter experts and tested on 5% of the sample at Debre Markos Comprehensive Specialized Hospital for applicability and data collection tool was revised accordingly. One day of training was given to the data collectors by the principal investigatorbefore the actual data collection. During data collection, close supervision and monitoring were conducted by the investigator.

**Data analysis**: Data were entered using EpiData software version 3.1 and cleaning, coding, and analysis was done using STATA software version 14.1. Variance inflation factor test was performed to detect the presence of multicollinearity between independent variables.

In the binary logistic regression analysis, crude odds ratio (OR) with a 95% CI was computed, and variables with a p-value≤0.25 were considered for multivariable analysis. In multivariable logistic regression, the adjusted odds ratio (AOR) with a 95% CI was computed, and a p-value <0.05 was used to declare variables as statistically significant associated factors of unplanned re-laparatomy. Results were expressed as percentages, means with standard deviation, median with its interquartile ranges (IQR), and adjusted odds ratio (AOR) along with its 95% confidence interval. Finally, the results were presented in text, tables, and figures.

**Ethical considerations**: The study was approved by the Research Review Committee of Debre Markos University (MHSC/R/C/C/D/01/05/16), and all procedure was done with the essence of beneficence, and data were kept confidential. There were no personal identifiers included from the patients' medical records during data extraction; thus it would not inflict any harm on the patients.

## Results

**Socio-demographic characteristics and medical conditions of study participants**: In this study, 384 patient charts were included. The mean (standard deviation) age of participants at the time of admission was 38.0±17.9 years ( ).

**Clinical characteristics of study participants**: Most of the study participants, 313 (81.5%), were referred from other institutions, and the median duration of symptoms was 3 days (IQR: (2, 5)). Of these patients, nearly two-third (65.6%) arrived on the same day of referral. About 181(47.1%) of the study participants had conditions related to bowel obstruction. The majority, (90.2%), were operated on the same day of admission. The median systolic and diastolic blood pressures at admission were 100 mmHg (IQR: (100, 120)) and 70 mmHg (IQR: (60, 70)), respectively.

Seventy-five (19.5%) had previous abdominal operation and were improved from their prior surgical conditions; 104(27.1%) patients had one or more comorbid medical conditions other than previous abdominal surgery. Among these, the most common comorbid conditions were cardiac disease, 24(6.2%),cancer, 19(4.9%), diabetes mellitus, 28(7.3%), respiratory disease, 22(5.7%), neurologic diseases 7(1.8%), and HIV/AIDS, 6(1.6%). The mean length of Hospital stay among re-operated patients was 12.1 days while the non-re-operated patients had stayed only 7.3 days in the hospital.

**Prevalence of early postoperative complications and need for re-laparatomy after emergency laparatomy**: Out of the 384 study participants, 204(53.1%) had one or more postoperative complications. Out of the 384 study participants, 33(8.6%) needed re-laparatomy in the early postoperative period. All re-laparotomies were unplanned and done while the patients were in the ward. A significant proportion, 224(58.3%), of the patients developed one or more postoperative complications in the early post-operative period (

**Associated factors of early re-laparatomy after emergency laparatomy**: In the bivariate analysis, pus or fecal contamination of the peritoneal cavity, longer operation time, and hypotension had a p-value less than 0.05 and were significantly associated with increased mortality after emergency laparatomy (p < 0.05). Duration of symptoms greater than 3 days, pus or fecal contamination of peritoneal cavity, Anesthesiology Society of America (ASA) status, previous surgery, tenderness, preoperative vasopressor use, bowel ischemia, intraoperative vasopressor use, blood loss and presence of comorbidities had a p-value less than 0.25 and were included in the multivariable logistic regression analysis.

In the multivariable logistic regression, the odds of re-laparatomy among patients with preoperative hypotension were more than three times higher [AOR: 3.3 (95% CI: (1.88, 9.40))]. The odds of having re-operation among patients with longer operation time (greater than 1 hour) was more than four times greater than those who had shorter procedure time [AOR: 4.5 (95% CI: (1.88, 10.64)].

## Discussion

The purpose of this study was to determine the prevalence of re-laparatomy and its associated factors after emergency laparatomy in the early (30 days) post-operative period of follow-up. In this study, the re-laparatomy rate was 8.6%. This is higher than the findings from a retrospective study in a teaching hospital reported by Abebe K., (13) in Ethiopia and an observational study from India (16), which reported re-laparotomy rates of 6.9% and 2.84% respectively. The difference may be related to the inclusion of elective surgical procedures in the analysis of these studies. Patients undergoing emergency laparotomy tend to be in a state of physiological derangement compared to elective intra-abdominal procedures (17,18).

However, the prevalence of re-laparatomy in this study (8.6%) is lower than the prevalence of re-laparatomy (17.2%) from Ethiopia done by Negussie et al. at Tikur Anbessa Specialized Hospital (7), Birhanu Y (15) (12.3%) and re-laparatomy rate (24%) by Scriba MF, et.al (14). These disparities may reflect differences in study subjects and disease incidence patterns. The mean age of patients who had re-laparatomy in the Western countries was higher than those in African and Indian reports (7, 8, 14, 16, 19-24), including this study. The high life expectancy and disease patterns, a more malignant condition in the Western world that tends to occur in older age, may have contributed to this discrepancy. Studies in South Africa and Congo (8, 14) reported mean age of 38.0 and 34.6 years which is similar to our finding (38.0 years). Moreover, re-operations in these studies were planned re-laparotomy (41%) unlike the findings in our study in which all re-laparatomies were un-planned (14).

The mean hospital stay was longer among patients who needed re-laparatomy. However, it was relatively shorter in this study than in other similar reports (25). This may be related to reduced mortality from close intensive care unit monitoring and follow-up in other study settings (16).

Patients who were hypotensive before initial surgery had an increased risk for un-planned reoperation [AOR: 3.3 (95% CI: (1.88, 9.40))]. This was also evidenced in other similar studies. This emphasizes the importance of recommendations from ERAS protocols designed to minimize the physiological impact and stress response of the surgical insult by patient and system optimization based on the patient's underlying health and comorbidities, and metabolic and immune status (26-28).

The odds of having re-operation among patients with longer operation time (greater than 1 hour) was more than four times greater than those who had shorter procedure time [AOR: 4.5 (95% CI: (1.88, 10.64)]. This finding is in line with a previous study by Birhanu Y. et.al. which reported odds of re-laparatomy of about 3.3(95% CI:1.40-7.41) (15). Similar findings were reported from a retrospective chart review by Mike K. Liang et.al (29). Longer operation time is associated with increased complications and mortality by increasing risks from prolonged anesthesia exposure and pulmonary complications ([30, 31). Prolonged operation time may also indicate the severity status of the underlying disease entity and the likelihood of a complicated postoperative course.

However, this study has some limitations. Firstly, it is a retrospective study that may have missed important patient-related parameters, including nutritional status. Secondly,it is a single-center study, and its external validity may be limited.

In conclusion,the re-laparatomy rate in this study was high. The findings from this study implied that patients who were hypotensive preoperatively and took a longer duration of procedure time (greater than 1 hour) were at a greater risk of re-laparatomy. This emphasizes a need for advocacy on patient resuscitation and monitoring.

## Figures and Tables

**Table 1:** Socio-demographic characteristics of study participants, Debre Markos Comprehensive Referral Hospital, Northwest Ethiopia, January 1, 2019 - December 31, 2022 (N=384)

Covariates	Category	Underwent Re-lapartomy		
		Yes N (%)	No N (%)	Total N (%)
Age (years)	4-20	3(4.1)	74(95.9)	77(20.1)
	21-30	6(6.9)	81(93.1)	87(22.6)
	31-40	9(13.6)	57(86.4)	66(17.2)
	41-50	6(10.3)	52(89.7)	58(15.1)
	51-64	5(9.6)	47(90.4)	52(13.5)
	65-80	4(9.1)	40(90.9)	44(11.5)
Sex	Male	23(9.7)	215 (90.3)	238(61.9)
	Female	10(6.8)	136(93.2)	146(38.1)

**Table 2:** Prevalence of postoperative complications after emergency laparatomy, Debre Markos Comprehensive Referral Hospital, Northwest Ethiopia, 2023 (N=384)

	Yes (percentage)*	No (percentage)
Surgical site infection	160 (41.7)	224 (95.8)
Intra-abdominal collection	94 (24.5)	290 (75.5)
Septic shock	29 (7.5)	355 (92.5)
Hospital-acquired pneumonia	84 (21.9)	300 (78.1)
Arrhythmia	11 (2.8)	373 (97.2)
Acute Kidney Injury	18 (4.7)	366 (95.3)
Deep Vein Thrombosis	14 (3.6)	370 (96.4)
Urinary Tract Infection	8 (2.1)	376 (97.9)

* The numbers may not add up to the total number of complications (204) as some may occur in a single individual.)

**Table 2:** Prevalence of postoperative complications after emergency laparatomy, Debre Markos Comprehensive Referral Hospital, Northwest Ethiopia, 2023 (N=384)

	Yes (percentage)*	No (percentage)
Surgical site infection	160 (41.7)	224 (95.8)
Intra-abdominal collection	94 (24.5)	290 (75.5)
Septic shock	29 (7.5)	355 (92.5)
Hospital-acquired pneumonia	84 (21.9)	300 (78.1)
Arrhythmia	11 (2.8)	373 (97.2)
Acute Kidney Injury	18 (4.7)	366 (95.3)
Deep Vein Thrombosis	14 (3.6)	370 (96.4)
Urinary Tract Infection	8 (2.1)	376 (97.9)

* The numbers may not add up to the total number of complications (204) as some may occur in a single individual.
